# Supplementary material for: Correlates of never testing for HIV among men who have sex with men in Nepal
Source: PLOS Glob Public Health. 2025 May 12;5(5):e0004432. doi: 10.1371/journal.pgph.0004432 (PMC12068604; doi:10.1371/journal.pgph.0004432)
Supplement: Attachment — Submitted filename: Rebuttal_Letter_Never_Testing RS.docx [file pgph.0004432.s004.docx]

4^th^ March 2025

Editor

PLOS Global Public Health

Re: PGPH-D-24-02775 “**Correlates of never testing for HIV among men who have sex with men in Nepal”**

Dear Editor Dr. Tsz Ho Kwan

Thank you for your comments and the reviewer’s feedback regarding our manuscript titled **“PGPH-D-24-02775; Correlates of never testing for HIV among men who have sex with men in Nepal.”** We have carefully revised the manuscript in response to the thorough and insightful observations we received. We are pleased to see interest in the topic and appreciate the constructive feedback.

We have addressed the comments point by point and have made corresponding changes in the manuscript in track changes (see below).

We hope this revised version will be suitable for publication in the PLOS Global Public Health.

Sincerely,

Dr. Roman Shrestha, Ph.D., MPH

**Reviewer #1:** The manuscript is sound and the conclusion is key to increasing knowledge in the field of study. Due to the nature of the variables, the bivariate and multivariate analyses utilized seem appropriate to the study. There are, however, some observations;

**#Reply:** Thank you for reviewing the manuscript and offering valuable suggestions that have enhanced the scientific rigor of this work.

**#Comment 1:**
**Abstract
The Information in the Abstract doesn't sufficiently highlight MSM engaging in transactional sex. If the findings among this group is the key outcome of the study, then it should clearly be highlighted the Abstract.**

**#Reply1:** Thank you for your suggestion. We have now highlighted transactional sex and other important variables in our results within the manuscript.

**#Comment 2:**
**What duration were they diagnosed with STIs? Is it a recent diagnosis? Will be great to qualify the "diagnosed with STIs" as "recently diagnosed" to enable adequate classification of the risk of acquiring the virus.**

**#Reply2:** Thank you for your question. This refers to individuals who have been diagnosed with any STI at any point in their lives. We have mentioned this in the methods section, and we have now updated the table and abstract accordingly in the manuscript.

**#Comment 3:**
**What percentage haven't tested due to Knowledge of testing using other kits, availability of kits in Health facilities, Community or Private sector, availability and accessibility to testing programs?**

**#Reply3:** Thank you for asking this question, but we did not measure knowledge of HIV testing kits or their availability and accessibility in testing programs. However, we have now acknowledged this limitation in our study. We have mentioned it as “*Third, we did not utilize a standardized questionnaire to assess participants' knowledge of HIV testing or the accessibility and availability of HIV testing programs.*”

**#Comment 4:**
**Main body
45; Why the focus on only Structural barriers? Is there specific role of Behavioral and Biomedical influence on the dependent variables? The statement of objective in the Abstract section doesn’t specify the structural factors as the key independent variables**.

**#Reply4:** Thank you for bringing this to our attention. Since our study does not solely focus on structural barriers, we have removed those sentences and revised them based on your suggestions to highlight the key outcomes of our study.

**#Comment 5:**
**50; If the focus is mainly on the influence of structural factors, then it should be stated in the main objective.**

**#Reply5:** Thank you. Since our study did not solely focus on structural barriers, we opted not to state this as a main objective.

**#Comment 6:**
**76; Any Global HIV Prevalence data among MSM? Would have been great to include this.**

**#Reply6:** Thank you for the suggestion. We have now included the HIV prevalence among MSM in the manuscript. The prevalence of HIV among MSM was found to be a median of 5% in Southeast Asia and 12.6% in Eastern and Southern Africa.

**#Comment 7:**
**79; It Would be nice to include data on HIV Prevalence among MSM in the region and/or Globally.**

**#Reply7:** Thank you for the suggestion; we have now included the HIV prevalence rates among MSM in the manuscript. The prevalence of HIV among MSM was found to be a median of 5% in Southeast Asia and 12.6% in Eastern and Southern Africa.

**#Comment 8:**
**163; What was the rationale for the 25yr mark? Is it in line with the cut off for the young persons” population In the Country**.

**#Reply8:** Thank you for raising this question. According to the United Nations (UN), anyone under the age of 25 is considered a young adult or youth. Additionally, other studies classify age in this way, as noted below;

1. <https://www.un.org/esa/socdev/documents/youth/fact-sheets/youth-definition.pdf>
2. Paudel K, Gautam K, Bhandari P, Wickersham JA, Dhakal M, Sharma S, Poudel KC, Ha T, Shrestha R. Suicidal ideation, plan, and attempt among men who have sex with men in Nepal: Findings from a cross-sectional study. PLOS Glob Public Health. 2023 Nov 22;3(11):e0002348. doi: 10.1371/journal.pgph.0002348. PMID: 37992034; PMCID: PMC10664887.
3. Wang, C., Wang, YJ., Tucker, J.D. *et al.* Correlates of HIV self-testing among female sex workers in China: implications for expanding HIV screening. *Infect Dis Poverty* **9**, 147 (2020). <https://doi.org/10.1186/s40249-020-00765-5>
4. Tsereteli N, Chikovani I, Chkhaidze N, Goguadze K, Shengelia N, Rukhadze N. HIV testing uptake among female sex workers and men who have sex with men in Tbilisi, Georgia. HIV Med. 2013 Oct;14 Suppl 3:29-32. doi: 10.1111/hiv.12065. PMID: 24033900.
5. Antos N, Flores R, Harawa N, Vecchio ND, Issema R, Fujimoto K, Khanna AS, Paola AD, Schneider JA, Hotton AL. Factors associated with HIV testing and treatment among young Black MSM and trans women in three jail systems. AIDS Care. 2023 Jan;35(1):123-130. doi: 10.1080/09540121.2022.2094312. Epub 2022 Jul 17. PMID: 35848452; PMCID: PMC10359835.

**#Comment 9:**
**245; It Would be great to compare the literature findings with the outcomes in the discussion section**.

**#Reply9:** In the paragraph where we missed comparing our study with other study outcomes, we addressed this issue in the discussion. We highly appreciate your feedback.

**Reviewer #2:** The manuscript presents an important analysis of HIV testing behaviors among men who have sex with men (MSM) in Nepal. The use of respondent-driven sampling (RDS) and multivariate analysis provides some insights into factors associated with never testing for HIV. The findings have the potential to inform targeted interventions aimed at increasing HIV testing among MSM in Nepal. However, there are several areas that require improvement, including clarity in the presentation of results, citation accuracy, methodological justifications, and addressing study limitations.

**#Reply:** Thank you very much for your constructive feedback. Your insights were extremely helpful in refining the manuscript.

**#Comment 1:**
**• Line 76 – The citation is inaccurate. The referenced source discusses Asia and the Pacific region, but the manuscript refers to global trends. Please correct this or provide a more appropriate global reference.**

**#Reply1:** Thank you for bringing this issue to our attention. We have included new references based on your suggestions.

**#Comment 2:**
**• Line 73 – The authors should cite the latest Global Burden of Disease (GBD) study and data for accuracy. A suitable reference is**: <https://www.thelancet.com/journals/lanhiv/article/PIIS2352-3018(24)00212-1/fulltext>.

**#Reply2:** Thank you for the suggestion, we cited your suggested citations in the manuscript.

**#Comment 3:**
**• Line 329 – The manuscript lacks a citation for the RDS methodology. A standard reference for RDS should be included.**

**#Reply3:** Thank you. We have cited the RDS methodology in the manuscript as a standard reference for RDS.

**#Comment 4:**
**• Line 163 – The age categorization (<25 vs. ≥25) needs justification. Why was this cutoff chosen? Would alternative classifications (e.g., using smaller intervals) provide more different results?**

**#Reply4:** Thank you for raising this question. According to the United Nations (UN), individuals under the age of 25 are considered young adults or youths. Additionally, other studies also classify age in this way, which are listed below:

6. <https://www.un.org/esa/socdev/documents/youth/fact-sheets/youth-definition.pdf>
7. Paudel K, Gautam K, Bhandari P, Wickersham JA, Dhakal M, Sharma S, Poudel KC, Ha T, Shrestha R. Suicidal ideation, plan, and attempt among men who have sex with men in Nepal: Findings from a cross-sectional study. PLOS Glob Public Health. 2023 Nov 22;3(11):e0002348. doi: 10.1371/journal.pgph.0002348. PMID: 37992034; PMCID: PMC10664887.
8. Wang, C., Wang, YJ., Tucker, J.D. *et al.* Correlates of HIV self-testing among female sex workers in China: implications for expanding HIV screening. *Infect Dis Poverty* **9**, 147 (2020). <https://doi.org/10.1186/s40249-020-00765-5>
9. Tsereteli N, Chikovani I, Chkhaidze N, Goguadze K, Shengelia N, Rukhadze N. HIV testing uptake among female sex workers and men who have sex with men in Tbilisi, Georgia. HIV Med. 2013 Oct;14 Suppl 3:29-32. doi: 10.1111/hiv.12065. PMID: 24033900.
10. Antos N, Flores R, Harawa N, Vecchio ND, Issema R, Fujimoto K, Khanna AS, Paola AD, Schneider JA, Hotton AL. Factors associated with HIV testing and treatment among young Black MSM and trans women in three jail systems. AIDS Care. 2023 Jan;35(1):123-130. doi: 10.1080/09540121.2022.2094312. Epub 2022 Jul 17. PMID: 35848452; PMCID: PMC10359835.

**#Comment 5:**
**• Line 173 – The manuscript states that participants were "grouped as multiple sex partners (yes/no)," but does not define the threshold for "multiple." The exact number of partners considered "yes" should be clarified.**

**#Reply5:** Thank you for asking this question, now we mentioned the definition of multiple sex partners in the manuscript as *“Individuals who engaged in sexual activity with more than one partner were classified as having multiple sexual partners.”*

**#Comment 6:**
**• Line 336-338 – The manuscript does not explain why the sample size was not increased. Was the sample size pre-determined?**

**#Reply6:** Thank you for asking this question. The sample size was based on previous studies conducted in this population. As this study is part of a larger research project, the sample size was predetermined. Following your suggestion, we have now cited our earlier studies in the methods section of the manuscript.

**#Comment 7:**
**• Line 230-232 – The manuscript can include confidence intervals in the text when discussing findings.**

**#Reply7:** Thank you for flagging this issue. Now, we have included confidence intervals in the text when discussing findings.

**#Comment 8:**
**• Line 274 – The findings in this paragraph are unclear. The discussion focuses on previous studies, but there is no clear linkage to the study's own results.**

**#Reply8:** Thank you for the suggestion. We have made the necessary changes to the manuscript and linked other studies with our own.

**#Comment 9:**
**• Line 272 – The phrase "This trust and emotional connection with their partner can also act as a barrier to regular testing like..." is incorrectly structured and should be rephrased for clarity**.

**#Reply9:** We appreciate your suggestion. We have rewritten this sentence now as *“This emotional connection with their partner may also serve as a barrier to regular HIV testing, as it could be perceived as a sign of distrust in the relationship”* in the manuscript.

**#Comment 10:**
**• Line 333 – The limitations section can address other limitations:**
o Due to the study’s cross-sectional design, causal relationships cannot be established.
o Potential limitations of RDS method.
o The face-to-face interview method may have influenced participants’ responses.

**#Reply10:** Thank you for the suggestions. We have now included this in the limitations of the manuscript. “*Third, we did not utilize a standardized questionnaire to assess participants' knowledge of HIV testing or the accessibility and availability of HIV testing programs. Fourth, the face-to-face interview format may have influenced participant’s responses due to social desirability bias. Fifth, the use of RDS may have introduced selection biases and limited the generalizability of the findings*”

**#Comment 11:**
**• Line 98 – The abbreviation ART is not defined upon first use.**

**#Reply11:** Thank you for the suggestion. We have now defined ‘ART’ in the manuscript the first time we use it.

**#Comment 12:**
**• Line 117-121 – The phrasing is repetitive and redundant.**

**#Reply12:** Thank you for bringing this issue to our attention. We have now removed the repetitive and redundant information from the manuscript.
