# Supplementary material for: Correlates of never testing for HIV among men who have sex with men in Nepal
Source: PLOS Glob Public Health. 2025 May 12;5(5):e0004432. doi: 10.1371/journal.pgph.0004432 (PMC12068604; doi:10.1371/journal.pgph.0004432)
Supplement: Attachment — Submitted filename: Rebuttal_Letter_Never_Testing RS _April.docx [file pgph.0004432.s005.docx]

4^th^ April 2025

Editor

PLOS Global Public Health

Re: PGPH-D-24-02775 “**Correlates of never testing for HIV among men who have sex with men in Nepal”**

Dear Editor Dr. Tsz Ho Kwan

Thank you for your comments and the reviewer’s feedback regarding our manuscript titled **“PGPH-D-24-02775; Correlates of never testing for HIV among men who have sex with men in Nepal.”** We have carefully revised the manuscript in response to the thorough and insightful observations we received. We are pleased to see interest in the topic and appreciate the constructive feedback.

We have addressed the comments point by point and have made corresponding changes in the manuscript in track changes (see below).

We hope this revised version will be suitable for publication in the PLOS Global Public Health.

Sincerely,

Dr. Roman Shrestha, Ph.D., MPH

**Reviewer #1: ABSTRACT**
**#Comment 1**

The abstract addresses the barriers to HIV testing, which is crucial for both HIV prevention and treatment.
• The aim of the study is clearly stated, and the focus on understanding the factors related to never having tested for HIV among MSM is both timely and important.
• The design is clearly stated, leveraging on a respondent-driven sampling (RDS), a robust method for sampling hidden populations like MSM, which enhances the generalizability of the findings to the broader MSM population in Nepal. The use of both bivariate and multivariate analyses is appropriate for understanding the relationships between participant characteristics and HIV testing.
• The abstract clearly states statistically significant findings (e.g., aORs and 95% CI) that identify factors associated with never testing for HIV, such as lack of awareness about HIV self-testing and PrEP, lack of engagement in transactional sex, and absence of daily internet access.

**#Reply 1:** Thank you so much for your detailed observation and comments. Your comments helped us shape this paper in this form.

**#Comment 2**
INTRODUCTION
• The introduction effectively situates the research within the broader global context of HIV and specifically highlights the vulnerable position of MSM. It is clear and informative in terms of the scale of the HIV epidemic.
• The introduction brings attention to the specific context of Nepal, which is crucial for understanding the regional challenges and the need for focused interventions.
• The rationale for the study, including the gap in the literature and the critical need for exploring the correlates of "never testing," is clearly articulated.
However, the final sentence of the introduction can be refined to reflect;
110. "Therefore, this study aims to identify factors associated with never testing for HIV among MSM in Nepal and to recommend targeted public health interventions to improve testing rates."

**#Reply 2:** Thank you for the suggestion. Now we have incorporated your feedback in the manuscript as per your suggestion as “Therefore, this study aims to identify factors associated with never testing for HIV among MSM in Nepal and to recommend targeted public health interventions to improve testing rates”

**#Comment 3**
METHODOLOGY
The methodology is robust and well-designed, using established techniques like RDS to reach the MSM population. Previous recommendations have been incorporated.
STATISTICAL ANALYSIS
191; It might be useful to briefly clarify why the Stata.SE Corp version 17.0 and RDSAT (RDS Analysis Tool 7.1) is chosen for the study.
193; It will be great to specify the continuous and categorical variable used for the descriptive analysis.
RESULTS
The Results section is well-structured and clear. The findings are presented logically with appropriate statistical backing, and the tables help summarize the data effectively.
However, it is recommended that the authors present a brief introductory sentence for the Results section and ensure consistency in terminology.

**#Reply 3:** Thank you for identifying the inconsistency in terminology in the manuscript. We have now ensured consistency throughout. Additionally, we have clarified the rationale for using RDSSTAT and STATA in the manuscript. Based on your suggestion, we have also specified continuous and categorical variables in the statistical analysis section.

**#Comment 4**
DISCUSSION
This Discussion section is well-written and provides a thorough interpretation of the findings. The section also incorporates the previous observations.
Consider linking the limitations of the study on the potential impact of the findings.
CONCLUSION
The conclusion is clear and summarizes the main findings effectively, while incorporating previous observations.

**#Reply 4**: Thank you for your nice words. Your comments helped in shaping the manuscript.
